# Diagnostic accuracy of research criteria for prodromal frontotemporal dementia

**DOI:** 10.1186/s13195-024-01383-1

**Published:** 2024-01-12

**Authors:** Alberto Benussi, Enrico Premi, Mario Grassi, Antonella Alberici, Valentina Cantoni, Stefano Gazzina, Silvana Archetti, Roberto Gasparotti, Giorgio G. Fumagalli, Arabella Bouzigues, Lucy L. Russell, Kiran Samra, David M. Cash, Martina Bocchetta, Emily G. Todd, Rhian S. Convery, Imogen Swift, Aitana Sogorb-Esteve, Carolin Heller, John C. van Swieten, Lize C. Jiskoot, Harro Seelaar, Raquel Sanchez-Valle, Fermin Moreno, Robert Jr. Laforce, Caroline Graff, Matthis Synofzik, Daniela Galimberti, James B. Rowe, Mario Masellis, Maria Carmela Tartaglia, Elizabeth Finger, Rik Vandenberghe, Alexandre Mendonça, Pietro Tiraboschi, Chris R. Butler, Isabel Santana, Alexander Gerhard, Isabelle Le Ber, Florence Pasquier, Simon Ducharme, Johannes Levin, Sandro Sorbi, Markus Otto, Alessandro Padovani, Jonathan D. Rohrer, Barbara Borroni, Genetic Frontotemporal dementia Initiative (GENFI), Annabel Nelson, Martina Bocchetta, David L. Thomas, Hanya Benotmane, Jennifer Nicholas, Rachelle Shafei, Carolyn Timberlake, Thomas Cope, Timothy Rittman, Andrea Arighi, Chiara Fenoglio, Elio Scarpini, Vittoria Borracci, Giacomina Rossi, Giorgio Giaccone, Giuseppe Di Fede, Paola Caroppo, Sara Prioni, Veronica Redaelli, David Tang-Wai, Ekaterina Rogaeva, Miguel Castelo-Branco, Morris Freedman, Ron Keren, Sandra Black, Sara Mitchell, Christen Shoesmith, Robart Bartha, Rosa Rademakers, Jackie Poos, Janne M. Papma, Lucia Giannini, Rick van Minkelen, Yolande Pijnenburg, Benedetta Nacmias, Camilla Ferrari, Cristina Polito, Gemma Lombardi, Valentina Bessi, Michele Veldsman, Christin Andersson, Hakan Thonberg, Linn Öijerstedt, Vesna Jelic, Paul Thompson, Tobias Langheinrich, Albert Lladó, Anna Antonell, Jaume Olives, Mircea Balasa, Nuria Bargalló, Sergi Borrego-Ecija, Ana Verdelho, Carolina Maruta, Catarina B. Ferreira, Gabriel Miltenberger, Frederico Simões do Couto, Alazne Gabilondo, Ana Gorostidi, Jorge Villanua, Marta Cañada, Mikel Tainta, Miren Zulaica, Myriam Barandiaran, Patricia Alves, Benjamin Bender, Carlo Wilke, Lisa Graf, Annick Vogels, Mathieu Vandenbulcke, Philip Van Damme, Rose Bruffaerts, Koen Poesen, Pedro Rosa-Neto, Serge Gauthier, Agnès Camuzat, Alexis Brice, Anne Bertrand, Aurélie Funkiewiez, Daisy Rinaldi, Dario Saracino, Olivier Colliot, Sabrina Sayah, Catharina Prix, Elisabeth Wlasich, Olivia Wagemann, Sandra Loosli, Sonja Schönecker, Tobias Hoegen, Jolina Lombardi, Sarah Anderl-Straub, Adeline Rollin, Gregory Kuchcinski, Maxime Bertoux, Thibaud Lebouvier, Vincent Deramecourt, Beatriz Santiago, Diana Duro, Maria João Leitão, Maria Rosario Almeida, Miguel Tábuas-Pereira, Sónia Afonso

**Affiliations:** 1 Neurology Unit, Department of Clinical and Experimental Sciences, University of Brescia, P.le Spedali Civili 1, 25123 Brescia, Italy; 2 Neurology Unit, Department of Neurological and Vision Sciences, ASST Spedali Civili di Brescia, 25123 Brescia, Italy; 3 Vascular Neurology Unit, Department of Neurological and Vision Sciences, ASST Spedali Civili di Brescia, 25123 Brescia, Italy; 4 Department of Brain and Behavioral Science, Medical and Genomic Statistics Unit, University of Pavia, 27100 Pavia, Italy; 5 Department of Molecular and Translational Medicine, University of Brescia, 25123 Brescia, Italy; 6 Department of Neurological and Vision Sciences, Neurophysiology Unit, ASST Spedali Civili di Brescia, 25123 Brescia, Italy; 7 Biotechnology Laboratory, Department of Diagnostics, ASST Spedali Civili di Brescia, 25123 Brescia, Italy; 8 Department of Medical and Surgical Specialties, Neuroradiology Unit, University of Brescia, 25123 Brescia, Italy; 9 Center for Mind/Brain Sciences-CIMeC, University of Trento, 38068 Rovereto, Italy; 10 Department of Neurodegenerative Disease, Dementia Research Centre, UCL Institute of Neurology, Queen Square, London, WC1N 3BG UK; 11 UK Dementia Research Institute at UCL, London, WC1E 6BT UK; 12 Department of Neurology, Erasmus Medical Centre, Rotterdam, 3015 GD The Netherlands; 13 Alzheimer’s Disease and Other Cognitive Disorders Unit, Neurology Service, Hospital Clinic, Institut d’Investigacións Biomèdiques August Pi I Sunyer, University of Barcelona, 08036 Barcelona, Spain; 14 Cognitive Disorders Unit, Department of Neurology, Donostia University Hospital, 20014 San Sebastian, Gipuzkoa Spain; 15 Neuroscience Area, Biodonostia Health Research Institute, 20014 San Sebastian, Gipuzkoa Spain; 16 Clinique Interdisciplinaire de Mémoire, Département des Sciences Neurologiques, CHU de Québec, and Facultéde Médecine, Université Laval, Quebec City, Québec G1V 0A6 Canada; 17 Center for Alzheimer Research, Division of Neurogeriatrics, Department of Neurobiology, Care Sciences and Society, Bioclinicum, Karolinska Institutet, 141 52 Solna, Sweden; 18 Unit for Hereditary Dementias, Theme Aging, Karolinska University Hospital, 141 52 Solna, Sweden; 19 Department of Neurodegenerative Diseases, Hertie-Institute for Clinical Brain Research and Center of Neurology, University of Tubingen, 72076 Tubingen, Germany; 20 Center for Neurodegenerative Diseases (DZNE), 72076 Tübingen, Germany; 21 Fondazione IRCCS Ca’ Granda, Ospedale Maggiore Policlinico, 20122 Milan, Italy; 22 Department of Biomedical, Surgical and Dental Sciences, University of Milan, 20122 Milan, Italy; 23 Department of Clinical Neurosciences and Cambridge University Hospitals NHS Trust and Medical Research Council Cognition and Brain Sciences Unit, University of Cambridge, Cambridge, CB2 7EF UK; 24 Sunnybrook Health Sciences Centre, Sunnybrook Research Institute, University of Toronto, Toronto, ON M4N 3M5 Canada; 25 Tanz Centre for Research in Neurodegenerative Diseases, University of Toronto, Toronto, ON M5S Canada; 26 Department of Clinical Neurological Sciences, University of Western Ontario, London, ON N6A 3K7 Canada; 27 Laboratory for Cognitive Neurology, Department of Neurosciences, KU Leuven, 3001 Leuven, Belgium; 28 Neurology Service, University Hospitals Leuven, 3000 Leuven, Belgium; 29 Leuven Brain Institute, KU Leuven, 3001 Leuven, Belgium; 30 Faculty of Medicine, University of Lisbon, 1649-028 Lisbon, Portugal; 31 Fondazione IRCCS Istituto Neurologico Carlo Besta, 20133 Milan, Italy; 32 Nuffield Department of Clinical Neurosciences, Medical Sciences Division, University of Oxford, Oxford, OX3 9DU UK; 33 Department of Brain Sciences, Imperial College London, London, SW7 2BX UK; 34 Neurology Service, Faculty of Medicine, University Hospital of Coimbra (HUC), University of Coimbra, 3004-561 Coimbra, Portugal; 35 Center for Neuroscience and Cell Biology, Faculty of Medicine, University of Coimbra, 3004-561 Coimbra, Portugal; 36 Division of Neuroscience and Experimental Psychology, Wolfson Molecular Imaging Centre, University of Manchester, Manchester, M20 3LJ UK; 37 Departments of Geriatric Medicine and Nuclear Medicine, University of Duisburg-Essen, 47057 Essen, Germany; 38 Cerebral Function Unit, Manchester Centre for Clinical Neurosciences, Salford Royal NHS Foundation Trust, Salford, M6 8HD UK; 39 Sorbonne Université, Paris Brain Institute - Institut du Cerveau - ICM, Inserm U1127, CNRS UMR 7225, AP-HP - Hôpital Pitié-Salpêtrière, 75013 Paris, France; 40 Centre de référence des démences rares ou précoces, IM2A, Département de Neurologie, AP-HP - Hôpital Pitié-Salpêtrière, 75013 Paris, France; 41 Département de Neurologie, AP-HP - Hôpital Pitié-Salpêtrière, 75013 Paris, France; 42 Univ Lille, 59000 Lille, France; 43 Inserm 1172, 59000 Lille, France; 44 CHU, CNR-MAJ, Labex Distalz, LiCEND Lille, 59000 Lille, France; 45 Douglas Mental Health University Institute, Department of Psychiatry, McGill University, Montreal, H3A 1A1 Canada; 46 McConnell Brain Imaging Centre, Montreal Neurological Institute, Department of Neurology & Neurosurgery, McGill University, Montreal, H3A 2B4 Canada; 47 Neurologische Klinik und Poliklinik, Ludwig-Maximilians-Universität, 80539 Munich, Germany; 48 Center for Neurodegenerative Diseases (DZNE), 81377 Munich, Germany; 49 Munich Cluster of Systems Neurology, 81377 Munich, Germany; 50 Department of Neurofarba, University of Florence, 50139 Florence, Italy; 51 IRCCS Fondazione Don Carlo Gnocchi, 50143 Florence, Italy; 52 Department of Neurology, University of Ulm, 89081 Ulm, Germany

**Keywords:** Prodromal, MCBMI, Frontotemporal dementia, Diagnostic criteria, Diagnostic accuracy

## Abstract

**Background:**

The Genetic Frontotemporal Initiative Staging Group has proposed clinical criteria for the diagnosis of prodromal frontotemporal dementia (FTD), termed mild cognitive and/or behavioral and/or motor impairment (MCBMI). The objective of the study was to validate the proposed research criteria for MCBMI-FTD in a cohort of genetically confirmed FTD cases against healthy controls.

**Methods:**

A total of 398 participants were enrolled, 117 of whom were carriers of an FTD pathogenic variant with mild clinical symptoms, while 281 were non-carrier family members (healthy controls (HC)). A subgroup of patients underwent blood neurofilament light (NfL) levels and anterior cingulate atrophy assessment.

**Results:**

The core clinical criteria correctly classified MCBMI vs HC with an AUC of 0.79 (*p* < 0.001), while the addition of either blood NfL or anterior cingulate atrophy significantly increased the AUC to 0.84 and 0.82, respectively (*p* < 0.001). The addition of both markers further increased the AUC to 0.90 (*p* < 0.001).

**Conclusions:**

The proposed MCBMI criteria showed very good classification accuracy for identifying the prodromal stage of FTD.

**Supplementary Information:**

The online version contains supplementary material available at 10.1186/s13195-024-01383-1.

## Introduction

Frontotemporal dementia (FTD) encompasses a clinically, genetically, and pathologically heterogeneous group of neurodegenerative disorders characterized by predominant degeneration of the frontal and/or temporal lobes. The clinical criteria have been defined based on presenting clinical symptoms, i.e., the behavioral variant of FTD (bvFTD) [1], which is associated with early behavioral and executive deficits; the agrammatic variant of primary progressive aphasia (avPPA), with progressive deficits in speech, grammar, and word output; and the semantic variant of PPA (svPPA), which is a progressive disorder of semantic knowledge and naming [2]. During the course of the disease, these phenotypes may change or overlap [3] and are often associated with motor features, including extrapyramidal symptoms, as in progressive supranuclear palsy (PSP) and corticobasal syndrome (CBS), or motor neuron disease (FTD-MND) [4, 5].

The initial phases of FTD, preceding overt dementia, are characterized by a potentially extended period during which biological (preclinical) and subsequently clinical (prodromal) alterations progressively accumulate, yet these stages remain inadequately delineated [6].

Recent advances in therapeutic strategies, particularly for monogenic disease, and the need for accurate counseling and guidance make the proper definition of these stages more compelling. In particular, several approaches have now been operationalized to define the prodromal stages of FTD, and it has been reported that biological markers, such as neurofilament light (NfL) or brain magnetic resonance imaging (MRI), are already altered in these early stages [7, 8].

The Genetic Frontotemporal Initiative (GENFI) Staging Group has recently proposed clinical criteria for the diagnosis of prodromal FTD, termed “mild cognitive and/or behavioral and/or motor impairment” (MCBMI) [6] to capture the entire disease complexity at presentation. The proposed MCBMI criteria include gradual and progressive cognitive and/or behavioral and/or motor changes compared to prior functioning and reported by the patient or informant, with preservation of independence in functional abilities of daily living, occurring along with one or more of the following symptoms: (a) objective evidence of a dysexecutive syndrome, occurring in isolation or associated with other cognitive changes, such as impaired social cognition; (b) language deficits; (c) behavioral changes including apathy, disinhibition, loss of empathy, compulsive behavior, and change in appetite; and (d) signs and symptoms of parkinsonism or motor neuron disease [6]. The validity of this set of symptoms in defining MCBMI-FTD needs to be further explored.

A genetically inherited disorder, most frequently due to variants in the *microtubule-associated protein tau (MAPT)*, *progranulin (GRN)*, or *chromosome 9 open reading frame72 (C9orf72)* genes [9, 10], may represent a privileged scenario to assess the MCBMI criteria accuracy.

These observations prompted the present study, aimed at validating the proposed set of criteria for MCBMI in the GENFI cohort, considering subjects carrying pathogenic FTD variants with mild clinical symptoms compared to a healthy control group composed of non-carrier family members. Moreover, we wanted to assess whether blood NfL levels or MRI data could improve diagnostic accuracy.

## Materials and methods

### Participants

From the GENFI cohort study, subjects carrying a pathogenetic FTD variant and non-carrier family members were recruited from research centers across Europe and Canada (www.genfi.org.uk).

All participants underwent the GENFI standardized assessment [11]. During the first visit, demographic characteristics of all participants were collected, as well as information regarding clinical background. As previously published, the years to expected onset were calculated as the difference between age at assessment and mean age at onset within the family [11, 12]. Despite the variability in correlation strength across genetic groups, with the strongest observed for *MAPT* and the weakest for *GRN*, this approach remains one of the most dependable methods currently available for estimating age at disease onset in mutation carriers [11–13]. A subgroup of patients nearing their estimated disease onset was identified as those with an estimated years to onset of < 5 years. While recognizing that the variability in estimating disease onset may condition this time frame, within our cohort, particularly among patients who transitioned to a fully symptomatic status at follow-up, this cutoff demonstrated that the majority of those who converted (77.8%) had an estimated disease onset of < 5 years. It is noteworthy that predicting precise conversion timelines in FTD is inherently challenging, as previously highlighted [14].

For the purpose of the present study, we included a consecutive sample of participants, carriers of an FTD pathogenic variant (*MAPT*, *GRN*, or *C9orf72*) with mild clinical symptoms and non-carriers as healthy controls (HC). In keeping with current literature and the aim of the present study, mild clinical symptoms were defined as a global CDR^®^ Dementia Staging Instrument plus National Alzheimer’s Coordinating Centre (NACC) behavior and language domains [15, 16] (CDR plus NACC FTLD) of 0.5 or a CDR plus NACC FTLD of 0 along with mild but significant motor symptoms. Unlike the global CDR score for which the memory domain is regarded as the primary domain and the others secondary, all eight domains of the CDR plus NACC FTLD are equally weighted in calculating the global CDR plus NACC FTLD score, and if any domain has a rating of 0.5 or if the maximum domain score is 1 and all other domains are 0, the global CDR plus NACC FTLD score is equal to 0.5 [17]. We did not include patients with a full FTD phenotype, thus with a global CDR plus NACC FLTD score of ≥ 1, according to the following scoring, as previously published by Miyagawa et al.: “If the maximum domain score is 2 or 3 and all other domains are 0, the global score is 1; if the maximum domain score occurs only once, and there is another rating besides 0, the global score is one level lower than the level corresponding to maximum impairment; if the maximum domain score occurs more than once, then the global score is that maximum domain score” [17].

Local ethics committees approved the study at each site, and all participants provided written informed consent. The study was conducted according to the Declaration of Helsinki.

### Assessment of MCBMI

MCBMI was assessed by the following: (a) trial making test [18], semantic (animals) and phonemic fluencies (letters FAS) [19] scores to assess executive functions; (b) the mini-social cognition and emotional assessment (mini-SEA), which is composed from a reduced and modified version of the *Faux-Pas* test, and a facial emotions recognition test [20] scores to assess social cognition; (c) Boston Naming [21] and modified Camel and Cactus test (mCCT) [22] scores to test language; (d) presence of apathy, disinhibition, loss of empathy, compulsive behavior, and change in appetite, as reported by caregiver (which were rated on a 5-point scale: 0 = absent, 0.5 = questionable/very mild, 1 = mild, 2 = moderate, and 3 = severe) to assess behavioral disturbances; and (e) presence of signs of parkinsonism or motor neuron disease as referred by caregiver, including dysarthria, dysphagia, tremor, slowness, weakness, gait disorder, falls, and functional difficulties using hands (which were rated on a 5-point scale: 0 = absent, 0.5 = questionable/very mild, 1 = mild, 2 = moderate, and 3 = severe) to assess extrapyramidal and motor neuron signs and symptoms. For further details and practical examples for each symptom and level of severity, we refer readers to Table S1 of Samra et al. [23].

### Neurofilament light quantification

In a subset of participants (*n* = 173), plasma was collected by venipuncture and centrifuged (2000*g*, 10 min, at room temperature). The serum was frozen at − 80 °C within 3 h after collection, shipped, and analyzed without any previous thaw–freeze cycle. We measured NfL levels in duplicates by single molecule array (Simoa) technique on the Simoa HD-X Analyzer (Quanterix, Lexington, MA, USA), using the NF-light Advantage kit for NfL [24] according to the manufacturer’s instructions (dilution: 1/4). All measurements had a coefficient of variation (CV) below 20%. Technicians were blinded to the genotypic and clinical status of the samples.

### MRI visual rating

A subset of participants (*n* = 297) underwent MRI at their local site. The protocol, designed to match across scanners as much as possible, included a volumetric T1-weighted scan, as previously published [11]. Visual rating of cerebral atrophy of the complete imaging dataset of all participants was performed, blind to all clinical and genetic information, by two trained raters (A.B. and E.P.). We adopted the 4-point scale evaluating both left and right anterior cingulate atrophy, evaluated on the first anterior slice where the corpus callosum becomes visible, which has been shown to be specific for FTD [25]. The selection of the anterior cingulate atrophy visual rating scale was grounded in its demonstrated efficacy and validation in discriminating FTD from Alzheimer’s disease, as well as its applicability across pathologically confirmed FTLD subtypes [26]. While data-driven approaches like voxel-based morphometry (VBM) can provide comprehensive insights into patterns of atrophy, the practical applicability of such methods in routine clinical practice can be limited due to the requirement of specialized software and expertise. Moreover, Harper et al. [25] demonstrated significant correlations between the visual rating scales and objective measurements of atrophy in the corresponding brain regions, including smaller frontal regions like the anterior cingulate, ensuring the reliability and validity of these scales in assessing regional brain atrophy. In contrast, visual rating scales, particularly those that are validated and recognized for their utility in distinguishing between neurodegenerative disorders, provide an accessible and applicable tool for clinicians, ensuring that the criteria can be readily implemented in patient assessments and diagnoses.

Images were rated in native space, in keeping with standard clinical reads. To aid rating consistency, reference images for the rating scale were provided to raters [25]. The mean values obtained by both raters were considered for analyses. Inter-rater reliability was determined using the intraclass correlation coefficient (ICC) (two-way random, absolute ICC), which was equal to 0.78, comparable to previous studies [25].

### Statistical analysis

Baseline demographic and clinical variables were compared across the groups using the Mann-Whitney *U* test for continuous variables or Fisher’s exact test for categorical variables. Considering that neuropsychological tests have substantial variability in performance below the normal range, tests were coded as “normal” or “abnormal” based on age-, sex-, and education-adjusted *z*-scores, with an impairment defined as *z* ≤ − 1.5, obtained from the healthy control group. Binomial logistic regressions were used to evaluate the predictive models and receiver-operating characteristic (ROC) curves constructed from the logistic scores. Areas under the curves (AUCs), including 95% confidence interval (CI) values, are reported. Sensitivities and specificities were computed at Youden’s *J* index thresholds. Positive predictive values (PPV) and negative predictive values (NPV) were computed; PPV was defined as the number of true positives/(number of true positives + number of false positives) while NPV was defined as the number or true negatives/(number of true negatives + number of false negatives). Given the multivariable nature of the model, specific cutoff values for individual measures are not obtainable, as changes in one predictor are considered in the context of all other variables in the model.

Statistical significance was assumed at *p* < 0.05, and *p* values were two-sided. Data analyses were carried out using SPSS, version 25.0 (IBM Corp).

### Data availability

All study data, including raw and analyzed data, and materials will be available upon reasonable request.

## Results

### Participant characteristics

A total of 398 participants were enrolled, 117 of whom were carriers of an FTD pathogenic variant (51 *C9orf72*, 44 *GRN*, 22 *MAPT*) in the MCBMI phase, while 281 were familial non-carriers. Demographic characteristics for both carriers and non-carriers are reported in Table 1. The groups appeared similarly distributed in sex, education, and handedness. The prodromal FTD group appeared slightly older (*p* = 0.016), but this difference of ~ 5 years (50 vs 45) was not considered clinically meaningful. Carriers showed significantly higher levels of plasma NfL (*p* < 0.001). Anterior cingulate cortex atrophy was significantly different between carriers and non-carriers (*p* < 0.001).

**Table 1:** Demographic and clinical characteristics of the prodromal FTD group and healthy control group

	**Prodromal carriers (*n* = 117)**	**Healthy non-carriers (*n* = 281)**	***p* value**
**Age, years**	50.1 (39.4–56.9)	44.7 (37.8–56.6)	0.016
**Sex, *n* female (%)**	70 (59.8)	158 (56.2)	0.578
**Education, years**	15 (12–16)	15 (12–16)	0.858
**Handedness (R:L:A)**	105:10:2	258:19:4	0.702
**CDR plus NACC - SOB**	0.5 (0.5–1.5)	0.0 (0.0–0.0)	
**Genetic status, *n* (%)**^b^			0.189
***GRN***	44 (37.6)	99 (35.2)	
***C9orf72***	51 (43.6)	116 (41.3)	
***MAPT***	22 (18.8)	59 (21.0)	
**Neuropsychological tests**			
**TMT-A**	25.0 (19.0–33.0)	24.9 (19.0–31.0)	0.468
**TMT-B**	61.0 (45.0–80.0)	57.1 (47.0–72.8)	0.437
**Semantic fluencies**	23.0 (20.0–28.0)	24.0 (20.0–27.0)	0.784
**Phonemic fluencies**	40.0 (30.0–49.8)	41.0 (31.0–50.0)	0.581
**Mini-SEA**	25.6 (24.0–27.0)	26.0 (24.0–27.0)	0.444
**Boston naming**	28.0 (26.0–29.0)	28.0 (27.0–29.0)	0.097
**Modified camel and cactus test**	30.4 (29.0–31.0)	30.4 (29.2–31.0)	0.953
**Plasma NfL, pg/mL**	9.7 (6.7–15.9)	7.4 (5.0–10.6)	< 0.001
**Anterior cingulate cortex**^a^	0.75 (0.25–1.25)	0.50 (0.00–0.50)	< 0.001

Data are median (interquartile range (IQR)) or *n* (%). *p* values were calculated by the Mann-Whitney *U* test, *χ*^2^ test, or Fisher’s exact test, as appropriate

*R* right-handed, *L* left-handed, *A* ambidextrous, *CDR plus NACC – SOB* CDR® Dementia Staging Instrument plus National Alzheimer’s Coordinating Centre behavior and language domains sum of boxes, *NfL* neurofilament light

^a^See text for details

^b^For healthy non-carriers, the number of participants in each genetic group represents healthy participants with a family member with that particular genetic variant

### Behavioral features

Behavioral symptoms for each group are reported in Table 2. The most frequently rated symptoms in the prodromal FTD group were apathy (21.4%), followed by disinhibition (17.9%), loss of empathy (14.5%), compulsive behavior (13.7%), and change in appetite (10.3%). All behavioral features were significantly different between the groups (all *p* < 0.001).

**Table 2:** Behavioral and motor features of the prodromal FTD group and healthy control group

	**Prodromal carriers (*n* = 117)**	**Healthy non-carriers (*n* = 281)**	***p* value**
**Behavior**			
Disinhibition	21 (17.9%)	4 (1.4%)	< 0.001
Apathy	25 (21.4%)	11 (3.9%)	< 0.001
Loss of empathy	17 (14.5%)	2 (0.7%)	< 0.001
Compulsive behavior	16 (13.7%)	3 (1.1%)	< 0.001
Change in appetite	12 (10.3%)	4 (1.4%)	< 0.001
*** ≥ 1 behavioral symptom***	51 (43.6%)	19 (6.8%)	< 0.001
**Cognitive**			
Camel and Cactus	20 (17.4%)	20 (7.2%)	0.005
TMT A	52 (44.8%)	18 (6.5%)	< 0.001
TMT B	18 (15.5%)	24 (8.6%)	0.049
Boston Naming	77 (66.4%)	21 (7.5%)	< 0.001
Semantic fluencies	21 (18.1%)	15 (5.4%)	< 0.001
Phonemic fluencies	21 (18.1%)	20 (7.2%)	< 0.001
Mini-SEA	15 (13.0%)	16 (5.8%)	0.022
*** ≥ 1 cognitive impairment***	85 (72.6%)	47 (16.8%)	< 0.001
**Motor**			
Dysarthria	6 (5.1%)	3 (1.1%)	0.022
Dysphagia	7 (6.0%)	2 (0.7%)	0.003
Tremor	7 (6.0%)	11 (3.9%)	0.428
Slowness	7 (6.0%)	3 (1.1%)	0.009
Weakness	16 (13.7%)	1 (0.4%)	< 0.001
Gait disorder	9 (7.7%)	5 (1.8%)	0.006
Falls	7 (6.0%)	1 (0.4%)	0.001
Difficulties using hands	9 (7.7 %)	0 (0.0%)	< 0.001
*** ≥ 1 motor symptom***	30 (25.6%)	15 (5.3%)	< 0.001

Data are *n* (%). *p* values were calculated by the *χ*^2^ test or Fisher’s exact test

We observed that nearly half (43.6%) of prodromal FTD presented with at least one behavioral symptom while only 6.8% of the control group did (*p* < 0.001).

### Neuropsychological assessment

The frequency of impairment at formal neuropsychological testing is reported in Table 2. We observed significantly more impaired scores in the prodromal FTD group compared to the healthy control group in nearly all neuropsychological tests. In particular, the Boston Naming Test was impaired in 66.4% of prodromal FTD patients, followed by the Trail Making Test Part A (44.8%). Semantic and phonemic fluencies were similarly altered in 18.1% of prodromal FTD patients. We observed that at least one cognitive test was impaired in up to 72.6% of prodromal FTD, compared to just 16.8% of healthy controls.

### Motor features

Motor symptoms for each group are reported in Table 2. The most frequently rated symptoms in the prodromal FTD group were weakness (13.7%), followed by gait disorder (7.7%), functional difficulties using hands (7.7%), dysphagia (6.0%), tremor (6.0%), slowness (6.0%), falls (6.0%), and dysarthria (5.0%). Of all motor symptoms, only tremor was not significantly different between the groups. We observed that one quarter (25.6%) of prodromal FTD presented with at least one motor symptom while only 5.3% of the control group did (*p* < 0.001).

### Classification accuracy of proposed criteria

We tested the diagnostic accuracy of the proposed criteria in discriminating prodromal FTD from healthy controls and subsequently adding information on plasma NfL and/or anterior cingulate cortex atrophy evaluated by visual rating scale.

**Fig. 1:**
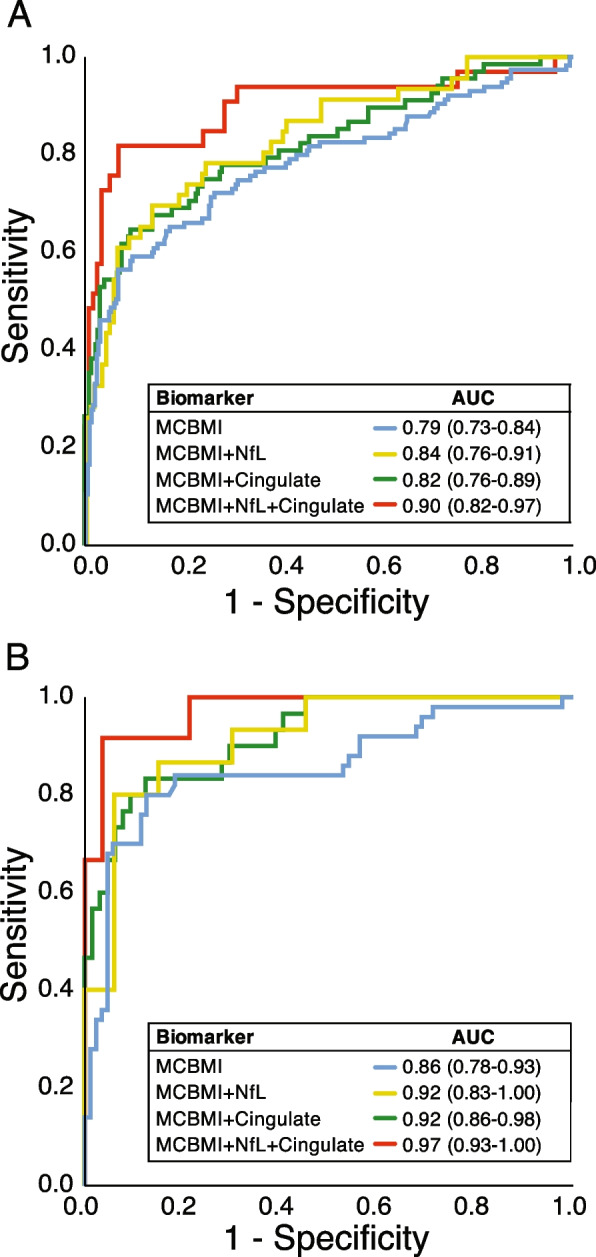
ROC curve analysis for differentiating prodromal FTD from healthy controls in **A** the whole group and in **B** participants with an estimated years to onset > − 5 years. ROC, receiver operating characteristics; AUC, area under the curve; NfL, neurofilament light; Cingulate, average left and right anterior cingulate cortex atrophy evaluated by visual rating scales

Considering the whole group, as shown in Fig. 1A and Table 3, the MCBMI criteria showed an AUC of 0.79 (95% CI 0.73–0.84), with a sensitivity of 56.5% and specificity of 93.4%. Diagnostic accuracy of behavioral, cognitive, and motor symptoms core features is reported separately in Additional file 1: Table S1.

**Table 3:** Diagnostic accuracy of the proposed criteria in classifying prodromal FTD from healthy controls

	**AUC (95% CI)**	**Sensitivity**	**Specificity**	**PPV**	**NPV**
**Whole group (*n* = 398)**					
Core criteria	0.79 (0.73–0.84)	56.5%	92.8%	76.5%	83.8%
Plasma NfL^a^	0.68 (0.59–0.77)	63.0%	66.1%	40.3%	83.2%
Anterior cingulate^b^	0.69 (0.62–0.77)	55.1%	77.6%	42.7%	85.1%
Core criteria + NfL^a^	0.84 (0.76–0.91)	69.6%	86.4%	65.3%	88.5%
Core criteria + anterior cingulate^b^	0.82 (0.76–0.89)	64.7%	90.6%	67.7%	89.4%
Core criteria + NfL + anterior cingulate^a^	0.90 (0.82–0.97)	81.8%	93.0%	77.1%	94.7%
**EYO < 5 years (*n* = 139)**					
Core criteria	0.85 (0.78–0.93)	80.0%	87.4%	78.4%	88.4%
Plasma NfL^c^	0.65 (0.49–0.81)	60.0%	69.7%	47.4%	79.3%
Anterior cingulate^d^	0.72 (0.61–0.83)	45.2%	89.2%	66.7%	77.3%
Core criteria + NfL^c^	0.92 (0.83–1.00)	80.0%	93.9%	85.7%	91.2%
Core criteria + anterior cingulate^d^	0.92 (0.86–0.98)	83.3%	87.5%	75.8%	91.8%
Core criteria + NfL + anterior cingulate^c^	0.97 (0.93–1.00)	91.7%	96.4%	91.7%	96.4%

*MCBMI* Mild cognitive and/or behavior and/or motor impairment, *AUC* Area under the curve, *PPV* Positive predictive value, *NPV* Negative predictive value, *NfL* Neurofilament light, *EYO* Estimated years to onset

^a^*n* = 173

^b^*n* = 297

^c^*n* = 48

^d^*n* = 96

Taken singularly, plasma NfL and anterior cingulate cortex atrophy showed similar accuracies (AUC of 0.68 [95% CI 0.59–0.77] with a cutoff of 8.53 pg/mL and 0.69 [95% CI 0.62–0.77], respectively). The addition of plasma NfL or anterior cingulate cortex atrophy to core clinical criteria similarly increased diagnostic accuracy (AUC of 0.84 [95% CI 0.76–0.91] and 0.82 [95% CI 0.76–0.89], respectively). The inclusion of both plasma NfL and anterior cingulate cortex atrophy to core clinical criteria showed the highest diagnostic accuracy, with an AUC of 0.90 (0.82–0.97), with a sensitivity of 81.8% and a specificity of 93.0% (see Fig. 1A and Table 3).

If we considered only participants approaching estimated disease onset (with estimated years to onset < 5 years, *n* = 139), the proposed MCBMI criteria showed higher accuracies, as reported in Fig. 1B and Table 3. The MCBMI criteria showed an AUC of 0.85 (95% CI 0.78–0.93), with a sensitivity of 80.0% and a specificity of 87.4%. Diagnostic accuracy of behavioral, cognitive, and motor symptoms core features in this group are reported separately in Additional file 1: Table S1.

The addition of plasma NfL or anterior cingulate cortex atrophy to the MCBMI criteria similarly increased diagnostic accuracy (AUC of 0.92 [95% CI 0.83–1.00] and of 0.92 [95% CI 0.86–0.98], respectively). The inclusion of both plasma NfL and anterior cingulate cortex atrophy to the MCBMI criteria showed the highest diagnostic accuracy, with an AUC of 0.97 (0.93–1.00), with a sensitivity of 91.7% and a specificity of 96.4%.

If we considered single genes separately, we observed comparable results (see Additional file 1: Table S2). In the *C9orf72* group, the MCBMI criteria added to plasma NfL and anterior cingulate cortex atrophy showed an AUC of 0.91 (0.82–0.99), with a sensitivity of 75.0% and a specificity of 95.5%. For *GRN*, we observed an AUC of 0.98 (0.94–1.00), with a sensitivity of 100.0% and a specificity of 85.0%, while for *MAPT*, we observed an AUC of 1.00 (1.00–1.00), with sensitivity and specificity of 100.0%.

Single ROC curves and cutoff values for each measure that was employed are reported in Additional file 1: Table S3.

## Discussion

In this study, we tested the proposed set of diagnostic criteria for mild cognitive and/or behavioral and/or motor impairment (MCBMI), which represents the prodromal stage of FTD. These criteria have shown good diagnostic accuracy in classifying MCBMI versus a group of non-carrier family members, with better specificity and negative predictive values than sensitivity.

The decision to include cognitive, behavioral, and motor symptoms in the definition of prodromal FTD stemmed from the evidence that all these symptoms, alone or in combination, may be observed in the prodromal stages [4, 11, 27–31]. Moreover, during the course of the disease, cognitive, behavioral, and motor symptoms may change or overlap [3], making the classification of a particular clinical syndrome particularly problematic in its infancy. Indeed, when we considered these items separately, we found that both behavior abnormalities, cognitive deficits, and even motor symptoms contributed to the definition of MCBMI. It is however true that additional cognitive tests and clinical features may allow to better refine classification accuracy and sensitivity of MCBMI-FTD. In the same view, considering neuropsychiatric symptoms in the framework of MCBMI [32] may possibly further improve its operational definition and neuropathological correlations.

As already reported in other prodromal neurodegenerative dementias [33, 34], we also aimed at assessing the add-on value of potential biological or imaging diagnostic markers. To this, we considered blood NfL measurements, already shown to be increased in both sporadic and genetic FTD, particularly during the conversion from the presymptomatic to symptomatic phase, even if not specific for the disease [7, 35, 36], and anterior cingulate cortex atrophy, which is scored easily by visual rating scales at single subject level and has been shown to be specific for FTD [25, 37, 38].

Interestingly, we observed that plasma NfL and anterior cingulate scores, taken singularly, have only modest accuracy in identifying prodromal FTD; however, when added to the MCBMI clinical criteria, both markers significantly increased diagnostic accuracy, and the highest classification was achieved when both markers were incorporated.

We also assessed the diagnostic accuracy in patients who were predicted to be approaching disease onset (with an estimated symptom onset < 5 years). In this case, nearly all classification models showed higher levels of diagnostic accuracy, possibly identifying participants approaching disease conversion.

In interpreting the ROC curves and the diagnostic accuracy when adding biomarkers, it is crucial to proceed with caution due to the potential for overfitting, especially given the specificity and rarity of our sample.

We observed similar results between different genetic groups (*C9orf72*, *GRN*, *MAPT*), thus possibly suggesting that these criteria could be accurate also in the sporadic presentations of disease. While there are documented similarities between familial and sporadic FTD [39–41], it is pivotal to acknowledge the existing literature that points to crucial differences in the underlying biology and pathology between these forms. Notable distinctions such as dipeptide repeats (DPR) pathology [42, 43] and increased tau co-pathology in *C9orf72* [44], lipofuscin presence in the retina of *GRN* carriers [45], and the heterogeneity of tau inclusion morphologies in *MAPT* versus sporadic tauopathies [46], as well as variations in biomarkers, including white matter hyperintensities in *GRN* FTD [47, 48] and altered CSF biomarker values in familial versus sporadic FTD [49], underscore the complexity and heterogeneity inherent in FTD. These differences necessitate a cautious approach in defining and understanding the prodromal state in both familial and sporadic FTD.

The MCBMI criteria, while conceptual, encompass a broad spectrum of symptoms and changes, including the vital consideration of gradual and progressive cognitive, behavioral, and motor changes compared to prior functioning, providing a subtle and dynamic characterization of early FTD. In contrast, the operationalization of these criteria, utilizing a CDR plus NACC FTLD score of 0.5, offers a standardized, quantifiable method for implementing the MCBMI criteria in practical settings. However, it is pivotal to note that the global CDR plus NACC FTLD, while instrumental in defining a stage of the disorder, does not inherently provide the tools to discriminate between early-stage FTD patients and healthy controls, underscoring the necessity of comprehensive, multidimensional criteria like MCBMI to accurately identify and characterize prodromal FTD.

This study brings further insights into the earliest phases of genetic FTD, joining the effort of other slightly different endeavors. Recently, the ALLFTD Consortium has operationalized the criteria for the prodromal behavioral variant of FTD (bvFTD), opting to use the term “mild behavioral and/or cognitive impairment in bvFTD (MBCI),” to acknowledge that both behavioral symptoms and cognitive impairment might be present during the disease prodrome [50], extending the previously published Rosovsky criteria to the earliest phases of the disease [1]. The bvFTD-MBCI and the MCBMI criteria address two key elements in the FTD field; the first with the attempt to capture a specific disease phenotype, while the second tries to identify the earliest phases of the global FTD spectrum. Both are reasonable and potentially useful depending on the clinical question, whether in relation to early-stage treatments, particularly for monogenic disease, that target the pathogenetic mechanisms of the disease regardless of the clinical phenotype. However, both approaches comply with a diagnostic tool rather than a screening test, reporting greater specificity than sensitivity.

We acknowledge that the present study entails several limitations. First, we did not include a control group with other neurodegenerative diseases, such as prodromal Alzheimer’s disease or non-neurodegenerative psychiatric disorders. This will be mandatory to confirm the validity of these criteria in real-world situations. Second, we did not perform a validation of the MCBMI criteria against a cohort that includes full phenotypes of FTD, as well as sporadic cases. While the criteria demonstrated validity in our specific cohort, further validation in cohorts encompassing a broader spectrum of FTD phenotypes and sporadic cases is crucial to ensure its applicability and validity in various clinical contexts. Third, while the scales used have shown good validity, it will be important in future studies to formally assess both intra- and inter-rater variabilities. Fourth, we acknowledge the limitation of not including premanifest disease carriers and not evaluating the stability of the prodromal status and phenoconversion to symptomatic syndromes which should be further assessed in future longitudinal studies.

The MCBMI criteria have demonstrated potential validity in identifying prodromal FTD within the confines of the present study, though further validation in diverse cohorts is essential to fully establish their validity and utility in clinical settings.

### Supplementary Information

**Additional file 1:** **Table S1.** Diagnostic accuracy of each domain of proposed MCBMI criteria in classifying prodromal FTD from healthy controls. **Table S2.** Diagnostic accuracy of proposed criteria in classifying prodromal FTD from healthy controls for each genetic group. **Table S3.** Diagnostic accuracy of each subdomain of proposed MCBMI criteria in classifying prodromal FTD from healthy controls.

## Data Availability

All study data, including raw and analyzed data, and materials will be available from the corresponding author, B.B., upon reasonable request.

## Abbreviations

AUC: Area under the curve
avPPA: Agrammatic variant of primary progressive aphasia
bvFTD: Behavioral variant frontotemporal dementia
C9orf72: Chromosome 9 open reading frame 72
CBS: Corticobasal syndrome
CI: Confidence interval
CDR plus NACC FTLD: CDR^®^ Dementia Staging Instrument plus National Alzheimer’s Coordinating Centre behavior and language domains
GENFI: Genetic Frontotemporal Initiative
GRN: Progranulin
HC: Healthy controls
FTD: Frontotemporal dementia
MAPT: Microtubule-associated protein tau
MCBMI: Mild cognitive and/or behavioral and/or motor impairment
MRI: Magnetic resonance imaging
NfL: Neurofilament light
PSP: Progressive supranuclear palsy
ROC: Receiver operating characteristic
svPPA: Semantic variant of primary progressive aphasia
