# Supplementary material for: Diagnostic accuracy of research criteria for prodromal frontotemporal dementia
Source: Alzheimers Res Ther. 2024 Jan 12;16:10. doi: 10.1186/s13195-024-01383-1 (PMC10785469; doi:10.1186/s13195-024-01383-1)
Supplement: Supplementary file 1 — Additional file 1: Table S1. Diagnostic accuracy of each domain of proposed MCBMI criteria in classifying prodromal FTD from healthy controls. Table S2. Diagnostic accuracy of proposed criteria in classifying prodromal FTD from healthy controls for each genetic group. Table S3. Diagnostic accuracy of each subdomain of proposed MCBMI criteria in classifying prodromal FTD from healthy controls. [file 13195_2024_1383_MOESM1_ESM.docx]

**Supplementary Table 1.** Diagnostic accuracy of each domain of proposed MCBMI criteria in classifying prodromal FTD from healthy controls.

|  | **AUC (95% CI)** | **Sensitivity** | **Specificity** | **PPV** | **NPV** |
| --- | --- | --- | --- | --- | --- |
| **Whole Group (n=398)** |  |  |  |  |  |
| Behaviour core | 0.69 (0.63 – 0.75) | 43.6% | 93.2% | 72.9% | 79.9% |
| Cognitive core | 0.58 (0.52 – 0.64) | 82.6% | 32.6% | 34.0% | 81.7% |
| Motor core | 0.59 (0.53 – 0.66) | 21.4% | 97.5% | 78.1% | 74.9% |
| **EYO < 5 years (n=139)** |  |  |  |  |  |
| Behaviour core | 0.73 (0.64 – 0.83) | 54.9% | 89.8% | 75.7% | 77.5% |
| Cognitive core | 0.62 (0.53 – 0.72) | 92.0% | 31.0% | 43.4% | 87.1% |
| Motor core | 0.62 (0.52 – 0.72) | 27.5% | 96.6% | 82.4% | 69.7% |

AUC = area under the curve; PPV = positive predictive value; NPV = negative predictive value; NfL = neurofilament light; EYO = estimated years to onset.

**Supplementary Table 2.** Diagnostic accuracy of proposed criteria in classifying prodromal FTD from healthy controls for each genetic group.

|  | **AUC (95% CI)** | **Sensitivity** | **Specificity** | **PPV** | **NPV** |
| --- | --- | --- | --- | --- | --- |
| ***C9orf72* (n=150)** |  |  |  |  |  |
| Core criteria | 0.78 (0.69 – 0.87) | 64.7% | 87.9% | 73.3% | 82.9% |
| Plasma NfL^*^ | 0.71 (0.58 – 0.83) | 90.0% | 45.5% | 45.5% | 90.9% |
| Anterior cingulate^†^ | 0.70 (0.58 – 0.81) | 59.3% | 76.8% | 45.7% | 85.1% |
| Core criteria + NfL^*^ | 0.90 (0.82 – 0.98) | 77.7% | 90.9% | 81.0% | 88.9% |
| Core criteria + anterior cingulate^†^ | 0.85 (0.74 – 0.95) | 77.8% | 87.8% | 67.7% | 92.3% |
| Core criteria + NfL + anterior cingulate^*^ | 0.91 (0.82 – 1.00) | 75.0% | 95.5% | 85.7% | 91.3% |
| ***GRN* (n=160)** |  |  |  |  |  |
| Core criteria | 0.79 (0.73 – 0.84) | 56.5% | 93.4% | 78.3% | 83.6% |
| Plasma NfL^‡^ | 0.69 (0.53 – 0.85) | 66.7% | 70.2% | 41.7% | 86.8% |
| Anterior cingulate^§^ | 0.59 (0.46 – 0.71) | 43.8% | 74.4% | 37.8% | 78.8% |
| Core criteria + NfL^‡^ | 0.92 (0.84 – 1.00) | 80.0% | 89.4% | 70.6% | 93.3% |
| Core criteria + anterior cingulate^§^ | 0.80 (0.69 – 0.91) | 65.6% | 91.1% | 72.4% | 88.2% |
| Core criteria + NfL + anterior cingulate^‡^ | 0.98 (0.94 – 1.00) | 100.0% | 85.0% | 68.4% | 100.0% |
| ***MAPT* (n=81)** |  |  |  |  |  |
| Core criteria | 0.90 (0.81 – 0.99) | 80.0% | 90.7% | 76.2% | 92.5% |
| Plasma NfL^‖^ | 0.61 (0.40 – 0.81) | 66.7% | 63.9% | 31.6% | 88.5% |
| Anterior cingulate^¶^ | 0.92 (0.81 – 1.00) | 70.0% | 98.0% | 87.5% | 94.2% |
| Core criteria + NfL^‖^ | 0.94 (0.86 – 1.00) | 88.9% | 94.1% | 80.0% | 97.0% |
| Core criteria + anterior cingulate^¶^ | 0.92 (0.80 – 1.00) | 88.9% | 95.8% | 88.9% | 97.9% |
| Core criteria + NfL + anterior cingulate^‖^ | 1.00 (1.00 – 1.00) | 100.0% | 100.0% | 100.0% | 100.0% |

AUC = area under the curve; PPV = positive predictive value; NPV = negative predictive value; NfL = neurofilament light; EYO = estimated years to onset.

^*^n=66; ^†^n=109; ^‡^n=62; ^§^n=122; ^‖^n=45; ^¶^ n=60.

**Supplementary Table 3.** Diagnostic accuracy of each subdomain of proposed MCBMI criteria in classifying prodromal FTD from healthy controls.

|  | **AUC (95% CI)** | **Cut-off score*** | **Sensitivity** | **Specificity** |
| --- | --- | --- | --- | --- |
| **Behaviour core** |  |  |  |  |
| Apathy | 0.59 (0.52 – 0.65) | 0.5 | 21.4% | 82.5% |
| Disinhibition | 0.58 (0.52 - 0.65) | 0.5 | 17.9% | 98.6% |
| Loss of empathy | 0.57 (0.50 – 0.63) | 0.5 | 14.5% | 86.2% |
| Compulsive behaviour | 0.56 (0.50 – 0.63) | 0.5 | 13.7% | 87.4% |
| Change in appetite | 0.54 (0.48 – 0.61) | 0.5 | 10.3% | 98.6% |
| **Cognitive core** |  |  |  |  |
| TMT-A | 0.52 (0.46 – 0.59) | 31.1 | 31.9% | 75.9% |
| TMT-B | 0.53 (0.46 – 0.59) | 63.5 | 47.7% | 61.2% |
| Semantic fluencies (animals) | 0.51 (0.45 – 0.57) | 23.0 | 60.3% | 46.2% |
| Phonemic fluencies (letters FAS) | 0.52 (0.46 – 0.58) | 53.5 | 17.2% | 87.9% |
| Mini-SEA | 0.52 (0.46 – 0.59) | 25.9 | 56.0% | 51.3% |
| Boston naming | 0.55 (0.49 – 0.62) | 28.9 | 44.8% | 65.5% |
| Modified Camel and Cactus test | 0.50 (0.44 – 0.57) | 29.5 | 73.7% | 29.6% |
| **Motor core** |  |  |  |  |
| Dysarthria | 0.52 (0.46 – 0.58) | 0.5 | 5.1% | 98.9% |
| Dysphagia | 0.53 (0.46 – 0.59) | 0.5 | 6.0% | 99.3% |
| Tremor | 0.51 (0.45 – 0.57) | 0.5 | 6.0% | 97.9% |
| Slowness | 0.53 (0.46 – 0.59) | 0.5 | 6.0% | 95.1% |
| Weakness | 0.57 (0.50 – 0.63) | 0.5 | 13.7% | 86.7% |
| Gait disorder | 0.53 (0.47 – 0.59) | 0.5 | 7.7% | 94.1% |
| Falls | 0.53 (0.47 – 0.59) | 0.5 | 6.0% | 94.4% |
| Functional difficulties using hands | 0.54 (0.48 – 0.60) | 0.5 | 7.7% | 92.3% |

AUC = area under the curve; TMT = trail making test; mini-SEA = mini-social cognition & emotional assessment.

*cut-off scores were obtained according to Youden’s *J* index.
